# Local membrane charge regulates β_2_ adrenergic receptor coupling to G_i3_

**DOI:** 10.1038/s41467-019-10108-0

**Published:** 2019-05-20

**Authors:** M. J. Strohman, S. Maeda, D. Hilger, M. Masureel, Y. Du, B. K. Kobilka

**Affiliations:** Department of Molecular and Cellular Physiology, Stanford University School of Medicine, Beckman Center Room B157, 279 Campus Drive, Stanford, CA 94305 USA

**Keywords:** Membrane lipids, G protein-coupled receptors, Hormone receptors

## Abstract

The β_2_ adrenergic receptor (β_2_AR) signals through both G_s_ and G_i_ in cardiac myocytes, and the G_i_ pathway counteracts the G_s_ pathway. However, G_i_ coupling is much less efficient than G_s_ coupling in most cell-based and biochemical assays, making it difficult to study β_2_AR−G_i_ interactions. Here we investigate the role of phospholipid composition on G_s_ and G_i_ coupling. While negatively charged phospholipids are known to enhance agonist affinity and stabilize an active state of the β_2_AR, we find that they impair coupling to G_i3_ and facilitate coupling to G_s_. Positively charged Ca^2+^ and Mg^2+^, known to interact with the negative charge on phospholipids, facilitates G_i3_ coupling. Mutational analysis suggests that Ca^2+^ coordinates an interaction between phospholipid and the negatively charged EDGE motif on the amino terminal helix of G_i3_. Taken together, our observations suggest that local membrane charge modulates the interaction between β_2_AR and competing G protein subtypes.

## Abstract (web summary)

In the healthy heart, the β_2_ adrenergic receptor (β_2_AR) signals through G_s_ and G_i_ proteins but the mechanism underlying G protein selectivity is not fully understood. Here, the authors show that membrane charge and intracellular cations modulate the β_2_AR−G_i3_ interaction.

## Introduction

A third of all FDA-approved pharmaceutical drugs function by modulating the activity of G-protein-coupled receptors (GPCRs)^1^, a large receptor superfamily. GPCRs catalyze the activation of heterotrimeric G proteins, which in turn initiate a multitude of signaling cascades that alter cellular function.

The β_2_ adrenergic receptor (β_2_AR) is a prototypical GPCR that mediates the fight-or-flight response. β_2_AR signals through both G_s_ and G_i_, and the dual G protein selectivity of β_2_AR is best characterized in heart muscle (cardiac myocytes). In healthy neonatal cardiac myocytes, epinephrine-stimulated β_2_AR immediately activates G_s_, increasing contraction rate, but after 10−15 min β_2_AR signals predominantly through G_i_ ^2^, which decreases the contraction rate. In cardiac myocytes, β_2_AR couples to both G_i2_ and G_i3_^3^. Of interest, G_i_ activation is impaired if β_2_AR internalization is blocked^4^. Also, G_i_ does not interact with a modified β_2_AR that internalizes but does not recycle to the plasma membrane^5^, or with WT β_2_AR that internalizes but is pharmacologically blocked from recycling^6^. Taken together, these observations demonstrate that β_2_AR−G_i_ interaction is regulated temporally and perhaps spatially.

During heart failure, a condition of chronic, progressive cardiac insufficiency, the G_i_ pathway counteracts some negative consequences of chronic G_s_ activation that exacerbate heart failure, namely apoptosis and structural and functional remodeling^7–9^. However, G_i_ activation reduces contractility, which can be problematic in certain models of heart failure^10^. More precise regulation of G_s_ and G_i_ activation is a therapeutic aim^9^.

While β_2_AR signals through both G_s_ and G_i_, the mechanism that initiates the G_s_-to-G_i_ switch in the healthy heart is not fully understood. Multiple biochemical mechanisms may play a role. PKA phosphorylation of β_2_AR has been reported to increase G_i1_ coupling in vitro^11^ and G_i_ coupling in HEK cells^12^; however, the G_i1_ subtype is not expressed in cardiac myocytes, and β_2_AR−G_i_ coupling is PKA independent in these cells^2^. In addition, GRK2 phosphorylation of β_2_AR has been suggested to increase G_i_ coupling^13^, but other investigators have reported that dephosphorylation is critical for β_2_AR recycling to the plasma membrane, and β_2_AR−G_i_ interactions^6^. Therefore, the mechanisms that modulate β_2_AR−G_i_ coupling remain unclear.

In vitro, negatively charged phospholipids stabilize an active conformation of the β_2_AR and enhance its affinity for the catecholamine isoproterenol^14^, but the effect of phospholipid charge on G protein coupling is unknown. Negatively charged lipids have previously been shown to facilitate β_1_AR−G_s_ interaction^15^, NTS1−G_q_^16^ interaction, CB_2_−G protein interaction^17^, and rhodopsin−G_t_^18^ interaction. In cardiac myocytes, β_2_AR activates G_s_ in T-tubules^19^, deep evaginations of the plasma membrane enriched in L-type calcium channels and negatively charged phospholipids^20^. β_2_AR activation of G_i_ may also occur in T-tubules, after internalization and recycling. However, trafficking events may alter the composition of phospholipids surrounding the β_2_AR. In addition, the β_2_AR−G_s_ signaling that occurs prior to β_2_AR−G_i_ signaling greatly increases the Ca^2+^ concentration near T-tubules^21^, which may alter the charge properties of the T-tubule lipids^22^.

Here we examine the effect of phospholipid charge on β_2_AR interactions with G_s_ and G_i3_. We find that negatively charged lipids enhance β_2_AR interaction with G_s_ and impair interaction with G_i3_. Further, Ca^2+^ and Mg^2+^ facilitate β_2_AR−G_i3_ interaction in negatively charged lipids. Our observations suggest that local membrane charge, tuned by intracellular cations, modulates β_2_AR interaction with G_i3_.

## Results

### Monitoring G protein coupling by fluorescence spectroscopy

**Fig. 1:**
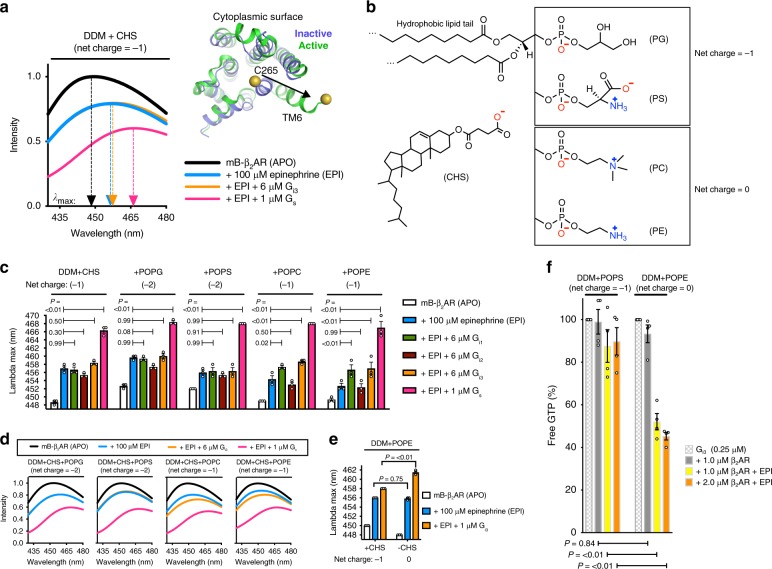
Negatively charged lipids inhibit β_2_AR−G_i_ coupling in detergent. **a** mB−β_2_AR emission spectra in DDM + CHS micelles (5:1 DDM:CHS mole ratio) in the absence of epinephrine (APO) and in the presence of epinephrine, ± G protein (G_s_ or G_i3_). Arrows point to the lambda max value, i.e. the wavelength where mB emission intensity is greatest. Inset shows how G_s_ coupling alters the structure of mB−β_2_AR, highlighting the change in position of monobromobimane (mB) at C265 of transmembrane 6 (TM6) (Inactive: PDB 5JQH^74^; Active: PDB 3SN6^69^). **b** Structures of CHS and phospholipids, with net charge indicated. **c** Effect of phospholipid on mB−β_2_AR interaction with G protein (G_i1_, G_i2_, G_i3_, and G_s_), read out as an increase in lambda max. Interaction was assessed in the absence of lipid (5:1 DDM:CHS mole ratio) and in the presence of POPG, POPS, POPC, or POPE (5:1:1 DDM:CHS:Lipid mole ratio). Multiplicity adjusted *P* values were computed by two-way ANOVA followed by Dunnett’s post hoc test between indicated groups. **d** Selected mB−β_2_AR emission spectra from panel (**c**). **e** Effect of CHS on mB−β_2_AR interaction with 1 μM G_i3_. Interaction was assessed in the presence of CHS (5:1:1 DDM:POPE:CHS mole ratio) or in the absence of CHS (5:1 DDM:POPE mole ratio). Multiplicity adjusted *P* values were computed by two-way ANOVA followed by Sidak’s post hoc test between indicated groups. **a**–**e** mB−β_2_AR concentration is 300 nM. Data are mean of three independent experiments. **f** β_2_AR-induced GTP turnover for G_i3_ (± 200 μM epinephrine, EPI) in DDM + POPS (5:1 DDM:POPS mole ratio) and in DDM + POPE (5:1 DDM:POPE mole ratio). Free GTP was assayed after 12 min. Luminescence signals were normalized relative to the condition with G_i3_ alone (see Supplementary Fig. 4). Data are mean ± s.e.m. of four independent experiments. Multiplicity adjusted *P* values were computed by two-way ANOVA followed by Sidak’s post hoc test between indicated groups. Source data are provided in the Source Data File

Epinephrine activates β_2_AR by partially stabilizing the conformation recognized by the G protein. This conformation is fully stabilized upon G protein coupling^23–25^. A feature of the G-protein-coupled conformation of β_2_AR is a 14 angstrom outward displacement of the cytoplasmic end of β_2_AR transmembrane segment 6 (TM6) (Fig. 1a). This conformation can be detected by fluorescence spectroscopy, using a modified β_2_AR labeled at the cytoplasmic end of TM6 with an environmentally sensitive fluorophore, monobromobimane (mB−β_2_AR, see Methods and ref. ^26^). Outward movement of TM6 affects the environmental polarity of mB, going from the hydrophobic receptor core to the solvent, decreasing the intensity of mB and increasing the wavelength where emission intensity is greatest (*λ*_max_), from ~447 to ~468 nm (Fig. 1a). We monitor *λ*_max_ to detect G protein coupling, as *λ*_max_ increases with coupling (Supplementary Fig. 1).

### Negatively charged lipids inhibit β_2_AR-G_i_ coupling

In the presence of epinephrine, we observe a change in intensity and *λ*_max_ of mB−β_2_AR following the addition of G_s_ in a detergent mixture containing *n*-dodecyl-β-d-maltopyranoside (DDM) and cholesteryl hemisuccinate (CHS) that is commonly used for biochemical study of GPCR/G protein complexes (Fig. 1a). In contrast, the coupling efficiency of mB−β_2_AR−G_i3_ was relatively weak (Fig. 1a). Next, we compared the coupling efficiency of mB−β_2_AR−G_i_ in DDM + CHS mixtures with different phospholipids incorporated (Fig. 1b–d). While we were unable to detect interactions of mB−β_2_AR with G_i1_, G_i2_ or G_i3_ in the presence of negatively charged lipids phosphatidylserine (POPS) and phosphatidylglycerol (POPG), we observed a weak interaction with G_i1_ and G_i3_ in neutral lipids phosphatidylethanolamine (POPE) and phosphatidylcholine (POPC) (Fig. 1c, d—G_i1_ and G_i3_ with epinephrine stabilizes more mB−β_2_AR in the active conformation than epinephrine alone). This result suggested that negatively charged lipids may repel G_i1_ and G_i3_ interaction with β_2_AR.

Indeed, we observed that negatively charged CHS decreased mB-β_2_AR-G_i3_ coupling (Fig. 1e), supporting our hypothesis that negatively charged lipids inhibit coupling. Since CHS is a nonphysiologic cholesterol analog, we omitted negatively charged CHS in subsequent experiments in order to isolate the effect of phospholipid charge on mB−β_2_AR−G_i_ interactions.

Given reports that PKA phosphorylation of β_2_AR increases β_2_AR−G_i_ interaction in vitro^11^, we also tested the effect of PKA phosphorylation, but no enhancement of mB−β_2_AR interaction with G_i1_, G_i2_, or G_i3_ was observed (Supplementary Fig. 2), suggesting that phosphorylation does not potentiate β_2_AR−G_i_ interaction under our experimental conditions, and that other mechanisms may enhance β_2_AR−G_i_ interaction.

In contrast to G_i1_ and G_i3_, we were unable to detect coupling to G_i2_ in any of the lipid-detergent mixtures (Fig. 1c), suggesting that the interaction is low affinity. Of interest, G_i1_ and G_i3_ share higher sequence identity than either subtype shares with G_i2_. To confirm the lack of coupling to G_i2_ observed by fluorescence analysis, we examined β_2_AR-stimulated G protein turnover of GTP in net neutral DDM + POPC for G_i2_ and G_i3_ (Supplementary Fig. 3). As expected from our fluorescence studies, we observed robust β_2_AR-induced GTP turnover for G_i3_, but only weak β_2_AR-induced GTP turnover for G_i2_. In contrast we observed strong coupling of G_i2_ to purified neurotensin receptor, indicating that the G_i2_ protein was functional. While both G_i2_ and G_i3_ couple to β_2_AR in cardiac myocytes^3^, the coupling efficiency using purified components is substantially different (see Discussion). We therefore focused on β_2_AR−G_i3_ coupling in further studies.

Next we examined how the lipid environment affects β_2_AR activation of G_i3_. In the presence of epinephrine, significantly more β_2_AR-induced GTP turnover was detected in DDM micelles containing POPE (net neutral lipid) than in DDM micelles containing POPS (net negative) (Fig. 1f). This effect on β_2_AR-mediated turnover was significant, even though the lipid:DDM mole ratio was only 1:5. POPE did not increase basal GTP turnover by G_i3_ in the absence of β_2_AR (Supplementary Fig. 4). Taken together, these results indicate that the charge property of phospholipids regulates G_i_ activation by β_2_AR.

### Ca^2+^ facilitates G_i3_ coupling in negatively charged lipids

**Fig. 2:**
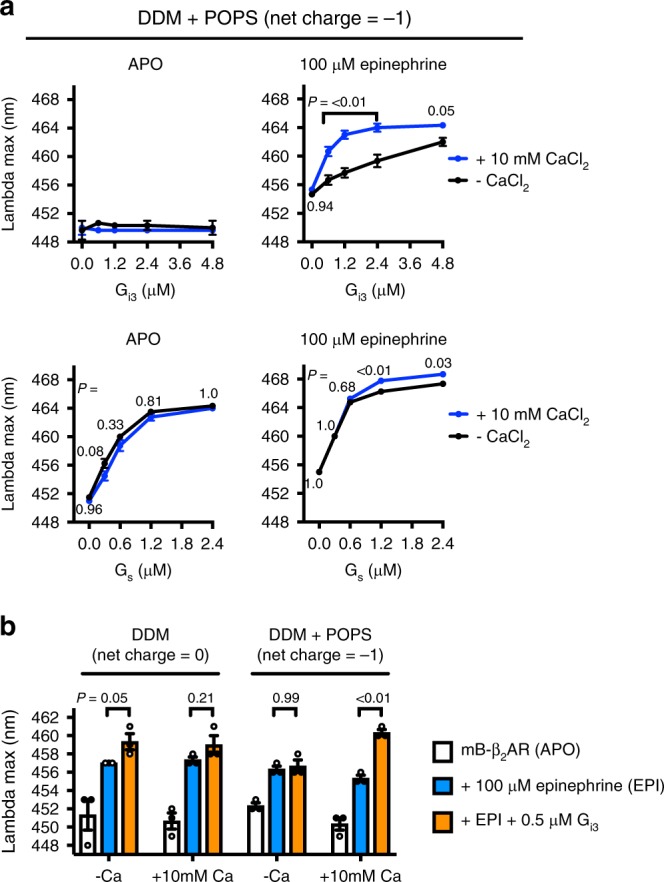
Ca^2+^ facilitates G_i3_ coupling in negatively charged phosphatidylserine. **a** The effect of 10 mM CaCl_2_ on mB−β_2_AR−G protein interaction (G_i3_ and G_s_) was examined in micelles containing 5:1 DDM:POPS (mole ratio). Data were collected in the absence (APO) and presence (100 μM) of epinephrine. mB−β_2_AR concentration is 250 nM. Data are mean ± s.e.m. of three independent experiments. Multiplicity adjusted *P* values were computed by two-way ANOVA followed by Sidak’s post hoc test between CaCl_2_ conditions. **b** The effect of 10 mM CaCl_2_ on mB−β_2_AR−G_i3_ interaction in DDM micelles ± POPS (i.e. DDM alone vs. 2.5:1 DDM:POPS mole ratio). mB−β_2_AR concentration is 250 nM. Data are mean ± s.e.m. of three independent experiments. Multiplicity adjusted *P* values were computed by three-way ANOVA followed by Sidak’s post hoc test between indicated groups. Source data are provided in the Source Data File

Ca^2+^, a ubiquitous second messenger, plays an important role in cardiac myocytes; Ca^2+^ waves, magnified by G_s_ activation, drive the cardiac myocyte contraction machinery. Recently Ca^2+^ was reported to regulate T-cell receptor activation by modulating the charge property of lipids^27^. Given that Ca^2+^ interaction with negative charge on phospholipids neutralizes the charge, we tested whether Ca^2+^ improves mB−β_2_AR−G_i3_ coupling efficiency in negatively charged DDM + POPS. Indeed, Ca^2+^ improved coupling efficiency in DDM + POPS micelles (Fig. 2a), and this effect required lipid (Fig. 2b). Moreover, Ca^2+^ had little effect on mB−β_2_AR−G_s_ interaction, implicating differences in G_s_ and G_i3_ surface charge.

### Ca^2+^ interacts with the amino terminal helix of G_i3_

**Fig. 3:**
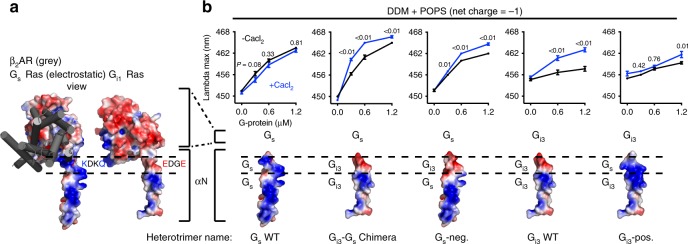
Ca^2+^ interacts with the amino terminal helix of G_i3_. **a** Membrane-facing surfaces of G_s_ and G_i1_ Ras domains, with β_2_AR shown in gray. The membrane-facing surface of G_i1_ was modeled by superimposing the Ras domain of G_i1_ (PDB: 1GP2^70^) onto the structure of G_s_ in complex with β_2_AR (PDB: 3SN6^69^). Red and blue signify negative and positive charge on G protein, respectively. Dashed lines highlight the region in αN where charge differs: The sequence is KDKQ in WT G_s_ vs. EDGE in WT G_i1_ (and in G_i2_, G_i3_). **b** mB−β_2_AR−G protein dose−response curves ± 10 mM CaCl_2_. Data were generated with the G protein depicted below the curves: mutations were made in αN and corresponding electrostatic models are shown. Epinephrine was not included in experiments titrating G_s_ WT, G_i3_-G_s_ Chimera, or G_s_-neg. to enhance the effect of CaCl_2_. Epinephrine (100 μM) was included in experiments titrating G_i3_ WT and G_i3_-pos. mB−β_2_AR concentration is 250 nM. Data are mean ± s.e.m. of three independent experiments. Multiplicity adjusted *P* values were computed by two-way ANOVA followed by Sidak’s post hoc test between CaCl_2_ conditions. Source data are provided in the Source Data File

Next, we sought to determine the mechanism by which Ca^2+^−POPS interactions increase mB−β_2_AR coupling to G_i3_ but not to G_s_. Given that the amino terminal helix (αN) of G protein is adjacent to the membrane when coupled to the β_2_AR^25^, and polybasic residues on G_s_ αN are known to facilitate membrane interaction^28^, we looked for a possible selectivity determinant within αN. Since αN of G_s_ and G_i_ are differentially charged (Fig. 3a), we first replaced αN of G_s_ with αN of G_i3_, creating a G_i3_-G_s_ chimera (Fig. 3b).

While Ca^2+^ does not promote mB−β_2_AR coupling to WT G_s_ (Fig. 3b), it did promote mB−β_2_AR coupling to the G_i3_-G_s_ chimera (Fig. 3b). Next, we compared the membrane-facing charge of G_s_ WT αN and G_i3_ WT αN. Structural analysis revealed that charge differed at the C terminal end of αN: G_i_ harbors a negatively charged motif (EDGE) at the position where G_s_ harbors a positively charged motif (KDKQ) (Fig. 3a). To examine whether this motif dictates a differential response to Ca^2+^, we constructed a G_s_ mutant (G_s_-neg.) containing the negatively charged motif of G_i3_ (KDKQ → EDGE). Ca^2+^ increased mB−β_2_AR interaction with this mutant (Fig. 3b), suggesting the EDGE motif is responsible for the effect of Ca^2+^ on G_i3_ αN. Taken together, our results imply that Ca^2+^ coordinates an interaction between the negatively charged EDGE motif on αN of G_i3_ and the headgroup of POPS. In the absence of Ca^2+^, like-charge repulsion decreases mB-β_2_AR coupling to G_i3_.

We also constructed a G_i3_ mutant (G_i3_-pos.) containing the positively charged motif of G_s_ (EDGE → KDKQ). The mutations only partially removed the effect of Ca^2+^ (Fig. 3b), indicating the effect of Ca^2+^ on G_i3_ extends beyond an effect on αN (see Discussion).

### Bilayer charge differentially affects G_s_ and G_i3_ coupling

Owing to their geometry and charge, phospholipids might induce changes in the size and shape of micelle assemblies which could also influence mB−β_2_AR−G protein interaction^29,30^. To examine the effects of phospholipids in a more native environment, and to restrict the size and shape of lipid ensembles, helping us isolate the effect of membrane charge, we reconstituted mB−β_2_AR into nanodisc bilayers and purified the nanodiscs to homogeneity using size-exclusion chromatography (Supplementary Fig. 5).

**Fig. 4:**
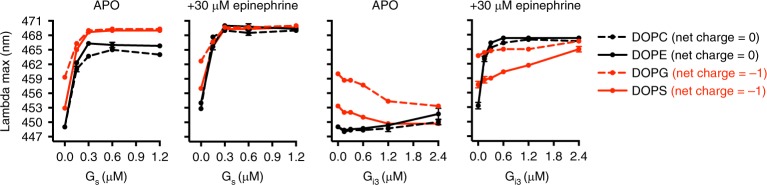
Bilayer charge differentially affects G_s_ and G_i3_ coupling. The effect of G_s_ (left) and G_i3_ (right) concentration on mB−β_2_AR fluorescence was examined in nanodisc bilayers of varying phospholipid composition (DOPC, DOPE, DOPG, DOPS). Epinephrine was omitted (APO) or included (30 μM). Interaction with G protein is read out as an increase in lambda max. The net charge of the phospholipid is indicated in parentheses. mB−β_2_AR concentration is 100 nM; maximum stoichiometry is 12:1 (for G_s_) and 24:1 (for G_i3_). Data are mean ± s.e.m. of three independent experiments. Source data are provided in the Source Data File

First, we compared the influence of lipid composition in the absence of Ca^2+^. Negatively charged bilayers (DOPG and DOPS bilayers), previously reported to increase agonist affinity^14^, expectedly red-shifted the emission spectra of unliganded mB−β_2_AR, suggesting these lipids stabilize an active conformation. Additionally, unliganded mB-β_2_AR and G_s_ could fully couple in negatively charged bilayers but not in neutral bilayers (Fig. 4), suggesting negatively charged lipids might facilitate signaling through G_s_. In contrast, negatively charged lipids (especially DOPS bilayers) decreased G_i3_ coupling to epinephrine-activated mB−β_2_AR (Fig. 4).

In fact, in negatively charged bilayers without epinephrine, G_i3_ unexpectedly blue-shifted the emission spectra of mB−β_2_AR. While this may indicate that G_i3_ stabilizes the β_2_AR in an inactive conformation in negatively charged lipids, it may represent a nonspecific interaction of inactive G_i3_ with the β_2_AR or the lipid bilayer.

### Cations promote G_i3_ coupling in negatively charged bilayers

**Fig. 5:**
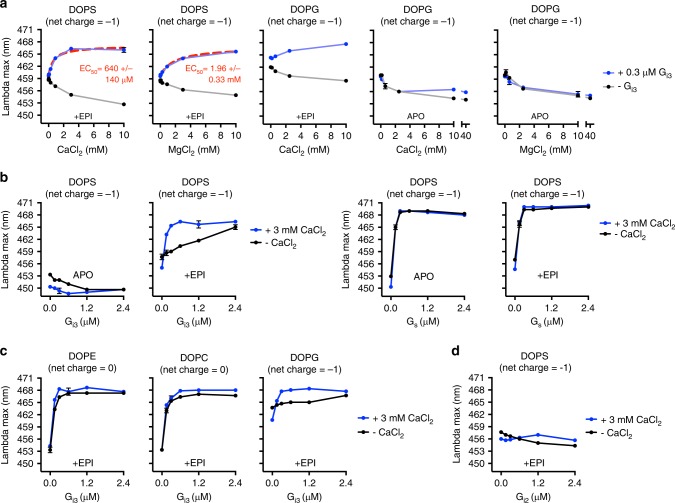
Ca^2+^ and Mg^2+^ facilitate G_i3_ coupling in negatively charged bilayers **a** The effect of CaCl_2_ and MgCl_2_ concentration on mB−β_2_AR fluorescence in DOPS and DOPG nanodisc bilayers was examined in the presence and absence of G_i3_. Epinephrine was included (30 μM) or omitted (APO). EC_50_ is mean ± s.e.m. **b** The effect of G protein concentration (G_i3_ and G_s_) on mB−β_2_AR fluorescence ± 3 mM CaCl_2_ was examined in DOPS nanodiscs in the absence (APO) and in the presence (EPI) of epinephrine (30 μM). **c** The effect of G_i3_ on mB−β_2_AR fluorescence ± 3 mM CaCl_2_ was examined in DOPE, DOPC, and DOPG nanodisc bilayers in the presence of 30 μM epinephrine (EPI). **d** The effect of G_i2_ concentration on mB−β_2_AR fluorescence ± 3 mM CaCl_2_ was examined in DOPS nanodisc bilayers in the presence of 30 μM epinephrine (EPI). **a**–**d** mB−β_2_AR concentration is 100 nM. The net charge of the phospholipid molecule is indicated in parentheses. Data are mean ± s.e.m. of three independent experiments. Source data are provided in the Source Data File

Next we examined the effect of Ca^2+^ and Mg^2+^. In the absence of G_i3_, both Ca^2+^ and Mg^2+^ reversed the active-state stabilizing effect of negatively charged DOPS and DOPG bilayers (Fig. 5a). Despite this, Ca^2+^ and Mg^2+^ increased mB-β_2_AR coupling to G_i3_ in negatively charged DOPS bilayers, but only Ca^2+^ was efficacious at concentrations below 1 mM (Fig. 5a). Ca^2+^ similarly affected mB−β_2_AR−G_i3_ interaction in negatively charged DOPG bilayers (Fig. 5a), but the magnitude of the effect in DOPG bilayers was less than observed in DOPS bilayers due to the higher baseline effect of DOPG on β_2_AR conformation.

We also compared the effect of Ca^2+^ on mB−β_2_AR interactions with G protein subtypes (G_s_ versus G_i3_) in DOPS bilayers. As observed in micelles, Ca^2+^ increased mB−β_2_AR coupling to G_i3_ but not to G_s_ (Fig. 5b). Ca^2+^ also improved mB−β_2_AR−G_i3_ coupling efficiency in negatively charged DOPG bilayers (Fig. 5c). However, incorporating Ca^2+^ did not enable detection of mB−β_2_AR interaction with the G_i2_ subtype of G_i_ (Fig. 5d, see Discussion).

While Ca^2+^ interacts with net negative PS and PG with relatively high affinity, it also interacts with the negatively charged phosphate group on net neutral PC^31^ and PE lipids^31,32^. In neutral DOPE and DOPC bilayers (Fig. 5c and Supplementary Fig. 6), Ca^2+^ only slightly enhanced mB−β_2_AR−G_i3_ interaction, which could be attributable to weaker Ca^2+^/DOPE and Ca^2+^/DOPC interactions that have been reported. Taken together, our observations provide biochemical evidence that local membrane charge can regulate β_2_AR−G protein interaction.

## Discussion

**Fig. 6:**
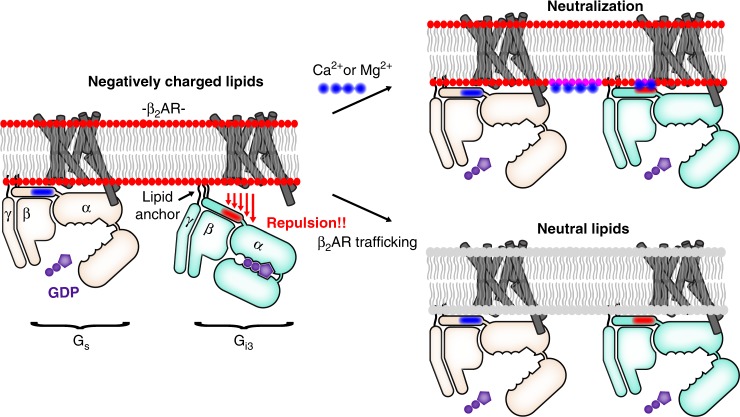
Membrane charge is a tunable modulator of β_2_AR−G protein interaction. Models depict epinephrine-bound β_2_AR. Left: In negatively charged lipids, β_2_AR−G_s_ coupling is efficient, but β_2_AR−G_i3_ coupling is relatively inefficient, in part because β_2_AR−G_i3_ attraction is countered by membrane-G_i3_ repulsion. Specifically, negatively charged lipids repel the negatively charged EDGE motif found on the amino terminal helix of G_i3_ (shown in red), a region that is positively charged in G_s_ (shown in blue). Right: Two mechanisms that neutralize membrane charge facilitate β_2_AR coupling to G_i3_. These mechanisms may play a role in G_s_-to-G_i_ switching in cardiac myocytes. Top right: Ca^2+^ and Mg^2+^ stabilize a like-charge interaction between the membrane and the EDGE motif. (Note that the effect of Ca^2+^ and Mg^2+^ may extend beyond an effect on αN positioning.) Bottom right: Epinephrine-stimulated β_2_AR traffics to membrane without negatively charged lipids

We observed that local membrane charge regulates β_2_AR−G protein interaction. Negatively charged membrane promotes β_2_AR−G_s_ coupling and suppresses β_2_AR−G_i3_ coupling. However, G_s_ bias is reduced in neutral membrane and in negatively charged membrane in the presence of divalent cations (see model in Fig. 6).

We have begun to explore the mechanism by which Ca^2+^ increases mB−β_2_AR coupling to G_i3_ in phospholipids. The effect of Ca^2+^ was largely dependent on charged groups on G_i3_, indicating Ca^2+^ doesn’t simply affect membrane structure. Although G proteins are membrane tethered via lipidation, the lipid anchor of G_i3_ is not sufficient for optimal interaction with β_2_AR in negatively charged bilayers, possibly due to repulsion of the carboxyl terminal end of the αN helix. We propose that Ca^2+^ helps orient the carboxyl terminal end of the αN helix of G_i3_ near the membrane, thereby facilitating β_2_AR−G_i3_ interactions. More specifically, we propose that Ca^2+^ facilitates the interaction of lipid with the negatively charged EDGE motif on the αN helix of G_i3_. Ca^2+^ may coordinate a like-charge interaction between the carboxylate groups on the G_i3_ EDGE motif and the phosphate group present on all lipids. In addition, Ca^2+^ may especially stabilize EDGE interaction with PS lipids by coordinating a like-charge interaction between the carboxylate groups on the G_i3_ EDGE motif and the carboxylate group on PS (not present on PG) (refer to structures in Fig. 1b), or Ca^2+^ might coordinate an intramolecular interaction between the phosphate group and the carboxylate group on PS, freeing the amino group (NH_3_^+^) on PS to interact with the carboxylate groups on G_i3_.

We have previously shown that negatively charged lipids, particularly PG, stabilize the β_2_AR in an active-like conformation as revealed by changes in mB−β_2_AR fluorescence and an increased affinity for agonists^14^. These effects are likely due to interactions between the lipids and positively charged amino acids on the β_2_AR. Here we observed that the effect of DOPG and DOPS on mB-β_2_AR can be reversed by both Ca^2+^ and Mg^2+^ (Fig. 5a). Yet, these divalent cations do not appear to reduce coupling to G_s_.

β_2_AR signals from caveolin-rich rafts^33,34^ within T-tubules^19^. While β_2_AR preferentially interacts with PG in insect cell membrane^14^, the phospholipid composition immediately adjacent to β_2_AR in T-tubules, and how it changes during β_2_AR trafficking, is currently unknown. Net-neutral PC and PE are the major phospholipids in T-tubules^35–37^. However, negatively charged PS is enriched in T-tubules relative to other membrane fractions (7.5−12.3% of total phospholipid)^35–39^. While cytosolic Ca^2+^ concentrations are typically less than 1 mM^40^, concentrations of Ca^2+^ in the mM range may be observed in cardiac myocytes.

Investigators have long speculated about the functional role of Ca^2+^ in the cleft between the T-tubule membrane (where β_2_AR is localized) and the juxtaposed sarcoplasmic reticulum (SR)^21,22,41^. During each action potential, extracellular Ca^2+^ flows into the cleft through L-type Ca^2+^ channels (LTCCs) on the plasma membrane and through ryanodine receptors (RyRs) on the sarcoplasmic reticulum^40^. Cleft Ca^2+^ concentrations spark to >100 μM in the absence of epinephrine and >1 mM^42,43^ following epinephrine stimulation, a consequence of G_s_ activation. Computational models show that negatively charged phospholipids buffer approximately half the Ca^2+^ released into the cleft^42^, and experiments have shown that 80% of inner-leaflet bound Ca^2+^ is bound to negatively charged phospholipids^44^. Additionally, biochemical investigations show that Ca^2+^ can cluster negatively charged PS^45^ and PIP_2_^46,47^ lipids.

β_1_AR and β_2_AR signaling through G_s_ alters calcium handling in the cardiac myocyte, and increases the magnitude of Ca^2+^ currents and Ca^2+^ transients, which stimulate cardiac contraction^40,48^. However, elevated Ca^2+^ concentrations also activate the Ca^2+^/calmodulin-dependent protein kinase II (CaMKII), which promotes apoptosis, and is implicated in structural remodeling that ultimately results in cardiac dysfunction^9,49–52^. Elevated Ca^2+^ also activates calcineurin, which exacerbates pathological hypertrophy^21^. For these reasons, β_1_AR-selective (and nonselective) beta blockers have proven to be efficacious medicines for treatment of heart failure^53^. However, β_2_AR, owing to its dual G_s_/G_i_ selectivity, is functionally distinct from the strictly G_s_-coupled β_1_AR, and several lines of evidence suggest β_2_AR−G_i_ signaling functions to keep G_s_ signaling in check via negative feedback: β_2_AR−G_i_ signaling occurs minutes after β_1_AR−G_s_ and β_2_AR−G_s_ signaling^2^, β_2_AR−G_i_ signaling suppresses changes in calcium handling^48,54^, and β_2_AR−G_i_ signaling is antiapoptotic^7,8^. While the mechanism that triggers β_2_AR−G_i_ signaling is unknown, our biochemical observations suggest elevated Ca^2+^ concentrations could trigger β_2_AR coupling to G_i3_. It is notable that overexpression of the Ca^2+^/sodium exchanger facilitates β_2_AR−G_i_ suppression of β_1_AR−G_s_ signaling^55^, and overexpression has been cited to increase the inward LTCC Ca^2+^ current^56^.

It is also notable that intracellular Ca^2+^ ^57,58^ and Mg^2+^ ^58^ concentrations rise during ischemia and rise even higher during reperfusion. Whether the rising concentrations facilitate β_2_AR-G_i_ signaling is unknown. However, β_2_AR−G_i_ signaling can reduce the extent of cardiac necrosis caused by ischemia and reperfusion^59^.

We were surprised that we did not observe mB−β_2_AR coupling to G_i2_ under conditions where we observed coupling to G_i1_ and G_i3_ (Fig. 1c, Supplementary Fig. 3). Although G_i2_ contains the αN EDGE motif, we did not observe mB−β_2_AR−G_i2_ interaction in the presence of Ca^2+^ (Fig. 5d). While the sequences of Gα_i1_ and Gα_i3_ are 94% identical, Gα_i2_ shares less sequence identity with Gα_i1_ and Gα_i3_, 88% and 86%, respectively. Supplementary Fig. 7 shows the location of the amino acid differences in G_i2_ relative to both G_i1_ and G_i3_ using the recent cryo-electron microscopy structure of the adenosine A1-G_i2_ complex (PDB: 6D9H^60^). It can be seen that these amino acids do not appear to interact directly with the receptor in the nucleotide-free complex. However, these amino acids may form weak interactions with the poorly ordered intracellular loop 3 that is not observed in the structure, or may interact with other domains of the receptor during complex formation. Previous studies provide evidence for at least one transient intermediate state in formation of the β_2_AR−G_s_ complex^61^. Both G_i2_ and G_i3_ couple to β_2_AR in cardiac myocytes^3^. However, during heart failure, G_i2_ expression is commonly upregulated^62–64^. In contrast, G_i3_ upregulation, while reported^65^, is less commonly observed. How selective G_i2_ upregulation influences β_2_AR−G_i_ signaling is not fully understood. Signaling through G_i2_ and G_i3_ could play different roles in cardiac physiology. It is possible that β_2_AR coupling to G_i3_ plays a role in preventing myocyte damage during transient ischemia or prolonged periods of adrenergic stimulation, such as during exercise. Both of these conditions would be associated with elevated cytosolic Ca^2+^. In contrast, β_2_AR coupling to G_i2_ may play a more prominent role in the failing heart.

G proteins are a large superfamily, grouped into four subfamilies (G_s_, G_i/o_, G_q/11_, G_12/13_) encoded by 16 different genes^66^. Each subfamily activates distinct signaling pathways, and functional effects are cell-type specific. Most GPCRs can signal through more than one G protein subfamily, and ongoing research attempts to identify mechanisms that regulate G protein selectivity within a cell^66^. Whether local membrane charge affects G_i_ interaction with other G_i_-coupled GPCRs is not currently known. In cardiac myocytes, β_2_AR signals from a PS-enriched, Ca^2+^-enriched microenvironment, which highlights the potential relevance of our biochemical observations. Additionally, the observation that Ca^2+^ sensing receptor (CaSR) switches from G_q_ to G_i_ after cytosolic Ca^2+^ increases^67^ is also potentially relevant to our findings. However, knowledge that Ca^2+^ facilitates G_i_ interaction with membrane is not sufficient to predict how Ca^2+^ might affect G_i_ interaction with other receptors.

In conclusion, we show that local membrane charge differentially modulates β_2_AR interaction with competing G protein subtypes (G_s_ and G_i_). This discovery expands our knowledge of mechanisms that regulate the G protein coupling selectivity of GPCRs.

## Methods

### G protein expression and purification

All G proteins were human heterotrimeric G proteins (Gα, Gβ_1_, Gγ_2_). The Gβ_1_ subunit contained an N-terminal 6xHis tag followed by a rhinovirus 3C protease site used for purification^61^. Gα_s_ was the short splice variant. The G_i3_-G_s_ chimera was created starting with Gα_s_, replacing residues 1−38 (the αN helix) with the equivalent region of Gα_i3_ (residues 1−31 of Gα_i3_)^68^. G_s_-neg. was a G_s_ mutant with residues 32−35 of Gα_s_ (KDKQ) replaced with the equivalent region in Gα_i3_ (EDGE). G_i3_-pos. was a G_i3_ mutant with residues 25−28 of Gα_i3_ (EDGE) replaced with the equivalent region in Gα_s_ (KDKQ). G protein was expressed in *Tni* insect cells (Expression Systems Cat. 94-002S) using two recombinant baculoviruses, a virus encoding Gα and a separate virus encoding both the Gβ_1_ and Gγ_2_ subunits. Following infection, cells were incubated for 48 h at 27 °C, harvested by centrifugation, and suspended in lysis buffer (10 mM Tris (pH 7.5), 100 μM MgCl_2_, 5 mM β-mercaptoethanol (BME), 10 μM GDP, and protease inhibitors). The membrane fraction was collected by centrifugation and solubilized using a Dounce homogenizer and buffer comprised of 20 mM HEPES (pH 7.4), 100 mM NaCl, 1% sodium cholate, 0.05% *n*-dodecyl-β-d-maltopyranoside (DDM), 5 mM MgCl_2_, 5 mM BME, 10 μM GDP, and protease inhibitors. The soluble fraction was isolated by centrifugation, G protein was captured on Sepharose Fast Flow (GE Healthcare) charged with nickel, and gradually exchanged into SEC buffer (20 mM HEPES (pH 7.4), 100 mM NaCl, 0.05% DDM, 100 μM TCEP, 10 μM GDP, 1 mM MgCl_2_). 3C protease was added to cleave G protein off the resin, and the G protein was dephosphorylated using calf intestinal alkaline phosphatase, antarctic phosphatase, and lambda protein phosphatase. Subsequently, G protein was isolated in SEC buffer using a Superdex 200 10/300 GL column (GE Healthcare). The main peak corresponding to heterotrimeric G protein was collected, concentrated, and frozen.

### Receptor expression, purification, and labeling

The β_2_AR construct was PN1^14^, where human WT β_2_AR (R16, Q27 variant) is modified to contain an N-terminal FLAG tag, a C-terminal rho-1D4 tag, a TEV protease cleavage site between V24 and T25, and a 3C protease cleavage site between G365 and Y366. Additionally, mutations were introduced to increase expression (M96T, M98T), to remove a glycosylation site (N187E) and to remove reactive cysteines (C378A, C406A). β_2_AR was expressed in *Sf9* insect cells (Expression Systems Cat. 94-001S) using recombinant baculovirus and media supplemented with 1 μM alprenolol. Cells expressing β_2_AR were harvested by centrifugation and suspended in lysis buffer (10 mM HEPES (pH 7.4), 1 mM EDTA, 1 μM alprenolol, and protease inhibitors). The membrane fraction was collected by centrifugation and solubilized using a Dounce homogenizer and buffer comprised of 20 mM HEPES (pH 7.4), 100 mM NaCl, 1% *n*-dodecyl-β-d-maltopyranoside (DDM), 0.03% CHS, 2 mM MgCl_2_, 1 μM alprenolol, and protease inhibitors. The soluble fraction was isolated by centrifugation and anti-FLAG (ATCC HB-9259) affinity chromatography was used to purify β_2_AR, remove alprenolol, and adjust detergent concentration (to 0.1% DDM, 0.01% CHS). Monobromobimane (mB, Thermo Fisher Scientific) labeling was then performed overnight with excess mB in the presence of 100 μM TCEP and the reaction was quenched with 5 mM l-cysteine before further purification. All β_2_AR preparations were functionally purified by alprenolol-Sepharose affinity chromatography and washed on an anti-FLAG column to remove ligand. The eluted β_2_AR was dialyzed in buffer comprised of 20 mM HEPES (pH 7.4), 100 mM NaCl, 0.1% DDM, and 0.02% CHS, concentrated, and dephosphorylated using lambda protein phosphatase. For experiments assessing phosphorylation, unphosphorylated β_2_AR was phosphorylated with protein kinase A in the presence of 2 mM ATP. β_2_AR was stored frozen. Phosphorylation was assessed using the Pro-Q Diamond Phosphoprotein Gel Stain (ThermoFisher Scientific), per the manufacturer’s instructions. Stained acrylamide gels were scanned with a Typhoon 9410 Imager (GE Healthcare). Recombinant human NTSR1 (residues 20−418) contained an A85^1.54^L mutation to increase expression. NTSR1 was purified from *Sf9* insect cells into buffer comprised of 20 mM HEPES (pH 7.5), 100 mM NaCl, 5% glycerol, 0.01% lauryl maltose neopentyl glycol (LMNG), 0.001% CHS, and 0.5 µM JMV 449, and stored frozen.

### Ligands

(−) epinephrine was purchased from Sigma (purity > 99%). JMV 449 was purchased from Tocris (purity 97.6%).

### Micelle composition

*n*-dodecyl-β-d-maltopyranoside (DDM), cholesteryl hemisuccinate (CHS), and 1-palmitoyl-2-oleoyl-sn-glycero-3-(PE,PC,PG,PS) lipids (Avanti Polar Lipids) were mixed in the indicated ratios and solubilized in chloroform. Chloroform was evaporated, and the films were re-suspended in 20 mM HEPES (pH 7.4), 100 mM NaCl.

### Fluorescence spectroscopy

In experiments examining mB-β_2_AR in micelles, mB−β_2_AR was preincubated (30 min room temperature) in micelle stock prior to dilution with other reaction components. pH 7.4 HEPES buffer (containing 100 mM NaCl, ± ligand, ± CaCl_2_ or MgCl_2_) and G protein were sequentially included. Mixtures were incubated 2.5–3.0 h at room temperature. Final mB−β_2_AR concentration was 100−300 nM. Emission spectra were read at 22 °C using a Fluorolog-3 spectrofluorometer (Horiba Jobin Yvon Inc.). (Bandpass = 4 nm; Excitation = 370 nm; Emission = 420−500 nm, collected in 1 nm increments). The raw S1c/R1c spectra were smoothed using Prism (GraphPad Software) (*n* = 15 neighbors, second-order polynomial). Lambda max is defined as the wavelength at which fluorescence emission is maximum. To determine the EC_50_, data were fit to the agonist vs. response model in Prism 7.0d software.

### GTP turnover

Where lipid environments were compared, samples were prepared as they were for fluorescence spectroscopy. Following β_2_AR incubation with G protein, 1 μM GTP + 5 μM GDP mixtures (final concentration) were added to initiate the GTP turnover reaction. Reaction buffer contained 20 mM HEPES (pH 7.4) and 100 mM NaCl. Where G_i2_ and G_i3_ were compared, the GTP turnover reaction was initiated by mixing a solution containing 10 μM GTP and ligand-bound receptor (4 μM β_2_AR + 800 μM epinephrine or 1 μM NTSR1 + 10 μM JMV 449) in equal volume with a solution containing 2.5 μM G protein, 20 μM GDP, 20 mM MgCl_2_, and 200 μM TCEP. At the indicated timepoints, the GTP remaining was assessed using the GTPase-Glo assay (Promega), which detects GTP using a luminescence readout. Luminescence was detected using a SpectraMax Paradigm plate reader equipped with a TUNE SpectraMax detection cartridge (Molecular Devices). Background luminescence was subtracted from experimental reactions.

### Statistics

Two-way and three-way ANOVA were performed using Graphpad Prism 7.0d.

### Electrostatic modeling

Structural views and mutant models were generated using PyMOL (Schrödinger, LLC). We selected rotomer positions that most closely matched those seen in PDB 3SN6^69^ (for the G_i3_-pos. model) and PDB 1GP2^70^ (for the G_s_-neg. model). Continuum electrostatics models were calculated using the APBS^71^ plugin (MG Lerner, University of Michigan, Ann Arbor) for PyMOL. Atomic charge and radii were calculated using the online PDB2PQR server^72^ (pH 7.4, PARSE force field, hydrogen bond optimization, clash avoidance).

### Nanodisc reagents

1,2-dioleoyl-sn-glycero-3-(PE,PC,PG,PS) lipids (Avanti Polar Lipids) were used because of their low phase transition temperature. Lipids were dissolved in buffer comprised of 20 mM HEPES (pH 7.5), 100 mM NaCl, 50 mM sodium cholate, 1 mM EDTA at 16.6 mM, and were sonicated on ice before use. The MSP belt was MSP1E3D1^73^. pMSP1E3D1 was a gift from Stephen Sligar (Addgene plasmid #20066). The protein was expressed in BL21(DE3) *E. coli* (Cat. 70235-3 Millipore Sigma), and cells were lysed with sonication in 10 mM Tris-HCl, 100 mM NaH_2_PO_4_, 6 M GuHCl, 1% Triton X-100 (pH 8.0). The soluble fraction was isolated by centrifugation and passed through a Sepharose Fast Flow (GE Healthcare) column charged with nickel. Immobilized protein was washed with buffer comprised of 10 mM Tris-HCl, 100 mM NaH_2_PO_4_, 6 M GuHCl, and 0.2% Triton X-100 (pH 7.0), and then washed with buffer comprised of 50 mM NaH_2_PO_4_, 300 mM NaCl, and 0.2% Triton X-100 (pH 8.0). The protein was eluted using 250 mM imidazole. Impurities were precipitated by two rounds of heating at 70 °C for 1 h; after each round, the soluble fraction was isolate by centrifugation. The final soluble fraction containing MSP1E3D1 was supplemented with 20 mM sodium cholate and purified further using a HiPrep 16/60 Sephacryl S-300 HR size-exclusion column (GE Healthcare) equilibrated with buffer comprised of 20 mM HEPES, 100 mM NaCl, 1 mM EDTA, and 20 mM sodium cholate. The main peak corresponding to MSP1E3D1 was collected, dialyzed in 20 mM HEPES, 100 mM NaCl, 1 mM EDTA, 5 mM sodium cholate, concentrated to 728 μM, and frozen.

### Nanodisc reconstitution

Nanodiscs were formed with one lipid type (i.e. 100% DOPS, DOPG, DOPE, or DOPC). Reconstitution was initiated by sequentially mixing water, 20× reconstitution buffer (400 mM HEPES (pH 7.5), 2 M NaCl, 20 mM EDTA), lipid, mB−β_2_AR, and MSP1E3D1. The volume of mB−β_2_AR stock added to the mixture was ~13% of the final mixture volume. MSP1E3D1 and mB−β_2_AR were added 1:10 (molar ratio). Lipid and MSP1E3D1 were added 35:1 (molar ratio). The mixture was incubated at 4 °C for 2 h. Subsequently, Bio-Beads SM-2 resin (107.1 mg per μmol lipid, Bio-Rad) were added (4 °C for 4 h) to remove detergent, which triggers the reconstitution of mB−β_2_AR in nanodisc bilayers. The soluble fraction was isolated by centrifugation. Bare nanodiscs were separated from nanodiscs containing mB−β_2_AR using anti-FLAG (ATCC HB-9259) affinity chromatography. The eluate was incubated with 5 mM EDTA at 4 °C for ≥ 1.5 h to remove divalent cations. Subsequently, samples were injected into a Superdex 200 10/300GL size-exclusion column (GE Healthcare) equilibrated in buffer comprised of 20 mM HEPES (pH 7.4), 100 mM NaCl, and the main peak corresponding to nanodisc mB−β_2_AR was harvested, concentrated, and frozen. The concentration of nanodisc mB−β_2_AR was approximated by SDS-PAGE, using detergent solubilized β_2_AR as a protein concentration reference standard.

### Reporting summary

Further information on research design is available in the Nature Research Reporting Summary linked to this article.

## Supplementary information

Supplementary Information

Reporting Summary

Source Data

## Data Availability

The source data underlying Figs. 1a, c−f, 2a, b, 3b, 4, 5a–d, and Supplementary Figs. 1a−c, 2a, b, 3a, b, 4, and 6 are provided as a Source Data File. pMSP1E3D1 is available from Addgene (#20066). All PDB files that were analyzed have been published before and can be obtained from the RCSB Protein Data Bank using the accession codes 5JQH, 3SN6, 1GP2, and 6D9H. A reporting summary for this Article is available as a Supplementary Information file. All other datasets supporting the findings of the study are available from the corresponding author on reasonable request.
