# Supplementary material for: Local membrane charge regulates β2 adrenergic receptor coupling to Gi3
Source: Nat Commun. 2019 May 20;10:2234. doi: 10.1038/s41467-019-10108-0 (PMC6527575; doi:10.1038/s41467-019-10108-0)
Supplement: Supplementary file 1 — Supplementary Information [file 41467_2019_10108_MOESM1_ESM.pdf]

Supplementary Figures

Local membrane charge regulates β_2 adrenergic receptor coupling to G_{i3}

Strohman et al.

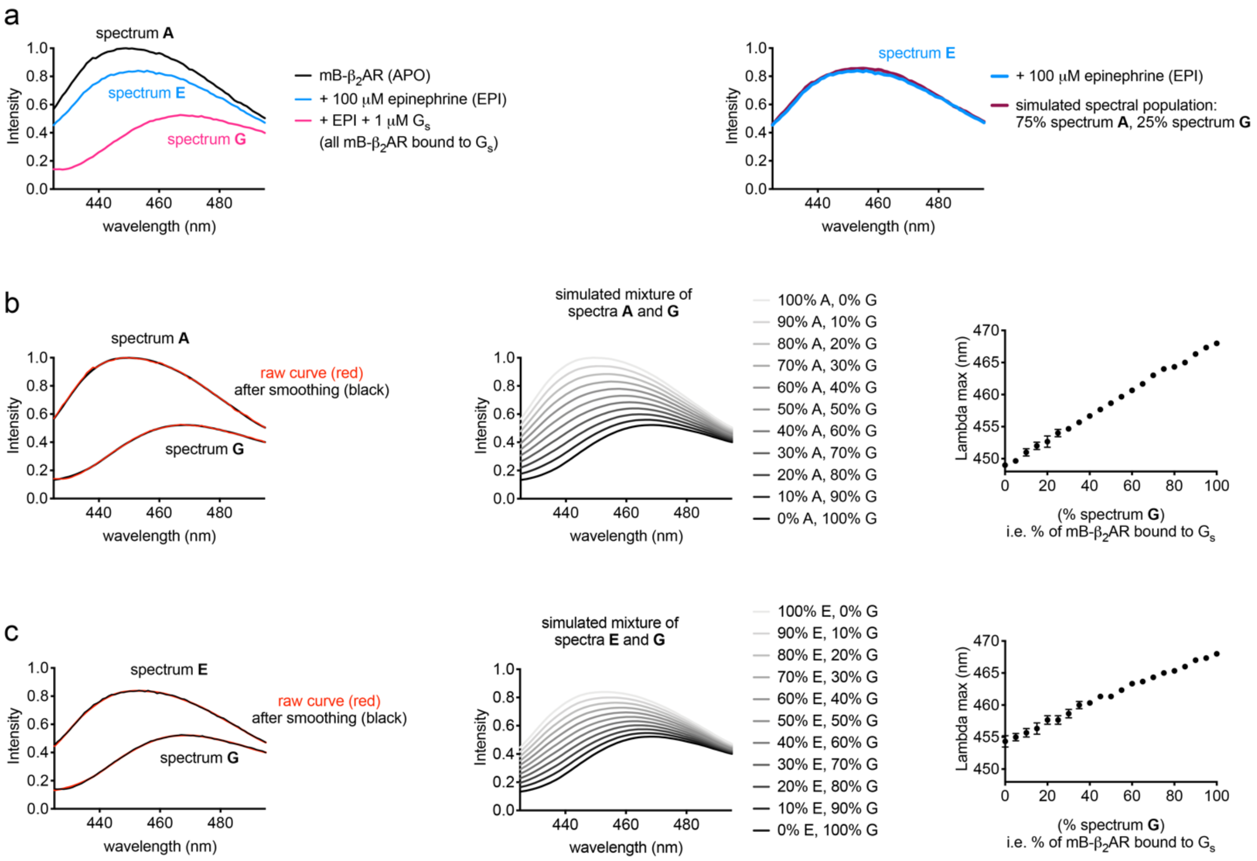

Supplementary figure 1. Simulated mB-β_2AR populations

This figure shows simulations to establish that, under the particular conditions of the experiments reported here, fluorescence emission maxima can be used to measure the fraction of G protein coupled β_2AR. (**a**) Left: Representative raw spectra from Figure 1d (DDM+CHS+POPC condition). Spectra are labeled as followed: **A** represents Apo mB-β_2AR, **E** represents mB-β_2AR bound to 100 μM epinephrine and **G** represents the mB-β_2AR-G_s complex. Right: Spectrum **E** relative to a simulated spectral population calculated as the weighted average intensity of spectra **A** and **G** ($Merged_{AG}$=0.75*$Intensity_A$+0.25*$Intensity_G$). (**b**) Left: Spectra **A** and **G** from panel A, with smoothed spectra underlaid in black. Middle: Simulated spectra with varying fractions of **A** and **G**. The simulated spectra were calculated as in panel A and were subsequently smoothed. Right: The λ_{max} has a linear correlation with the fraction of spectrum **G**. Data represent mean +/- s.e.m from three simulations based on spectra from three independent experiments. (**c**) Left: Spectra **E** and **G** from panel A, with smoothed spectra underlaid in black. Middle: Simulated spectra with varying fractions of **E** and **G**. The simulated spectra were calculated as in panel A and were subsequently smoothed. Right: The λ_{max} has a linear correlation with the fraction of spectrum **G**. Data represent mean +/- s.e.m from three simulations based on spectra from three independent experiments. Source data are provided in the Source Data File.

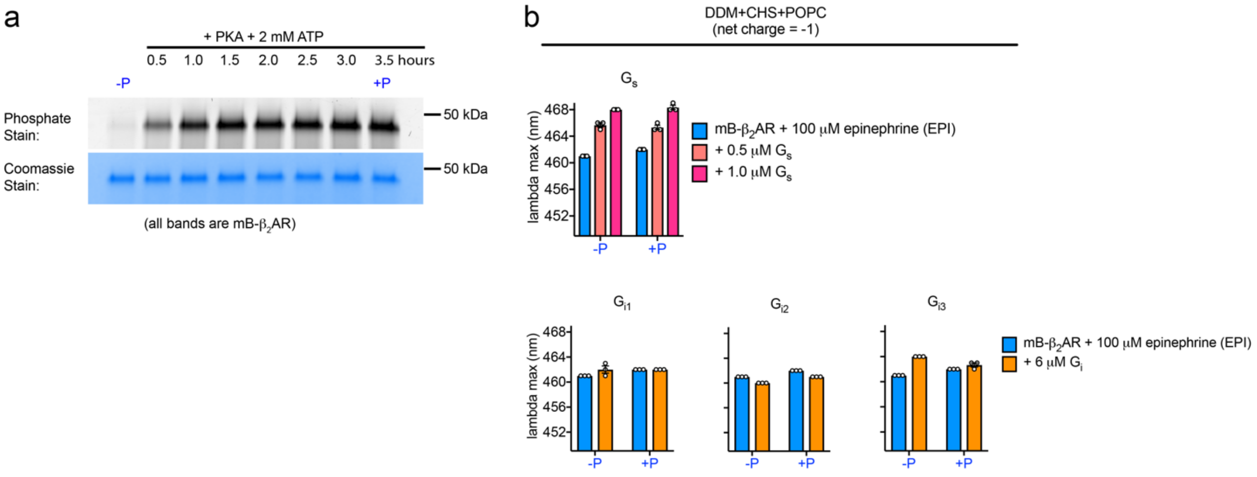

Supplementary figure 2. Effect of PKA phosphorylation on mB-β_2AR interaction with G_i (a) Phosphorylation status of mB-β_2AR before and every 0.5 hours after the addition of protein kinase A (PKA) and excess ATP (2 mM). -P indicates the unphosphorylated mβ-β_2AR used in Panel B; +P indicates the phosphorylated mβ-β_2AR used in Panel B (b) Effect of PKA phosphorylation on G_{i1}, G_{i2}, $G_{i3,}$ and G_s interaction with mB-β_2AR in the presence of epinephrine. Measurements were performed in DDM+CHS+POPC mixtures (5:1:1 DDM:CHS:POPC mole ratio). mB-β_2AR concentration is 300 nM. Data are mean +/- s.e.m of three independent experiments. Source data, including uncropped images, are provided in the Source Data File.

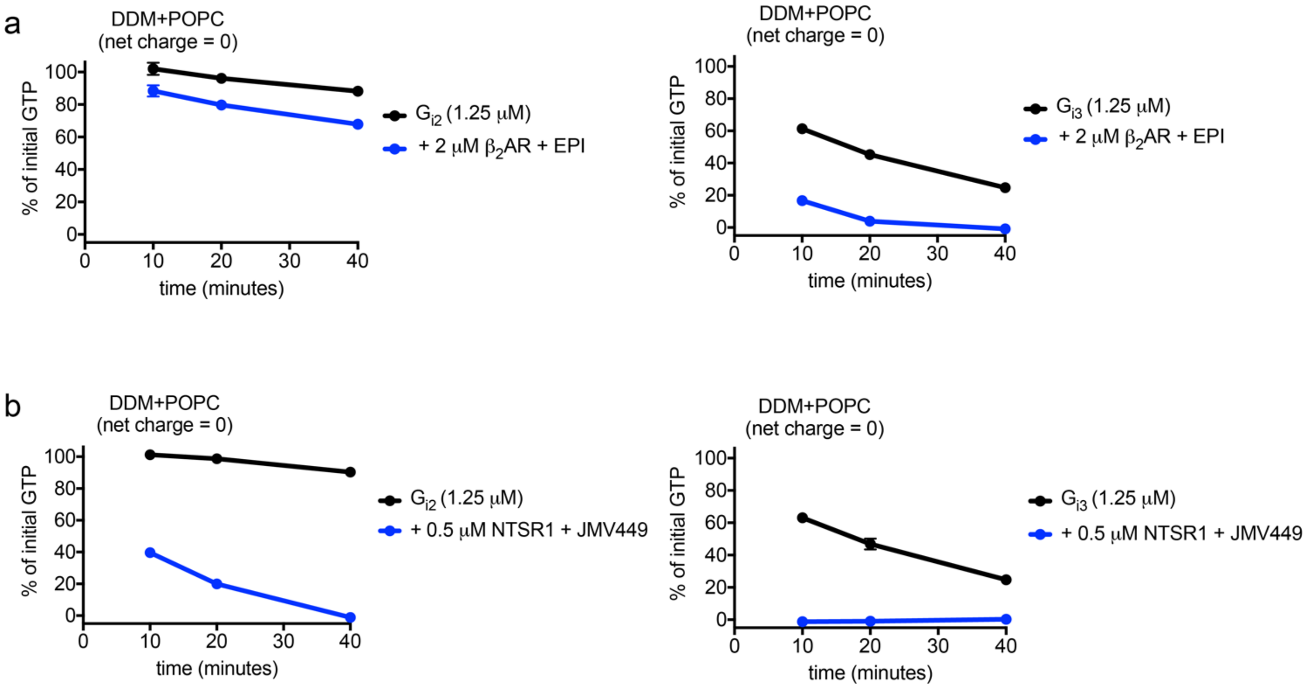

Supplementary figure 3. GTP turnover activity of G_{i2} (left) and G_{i3} (right) in DDM+POPC (5:1 DDM:POPC mole ratio) mixed micelles, in the absence or presence of ligand-stimulated $β_2$AR (panel A) and NTSR1 (panel B), using the ligand epinephrine (EPI) and JMV 449, respectively. Data are mean +/- s.e.m of three independent experiments. Source data are provided in the Source Data File.

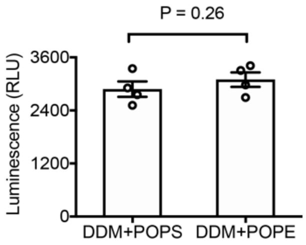

Supplementary figure 4. Luminescence signals from the Fig. 1f condition with G_{i3} alone (0.25 μM). Data are mean +/- s.e.m of four independent experiments. Statistical significance was determined using a two-sided Student's *t*-test. Source data are provided in the Source Data File.

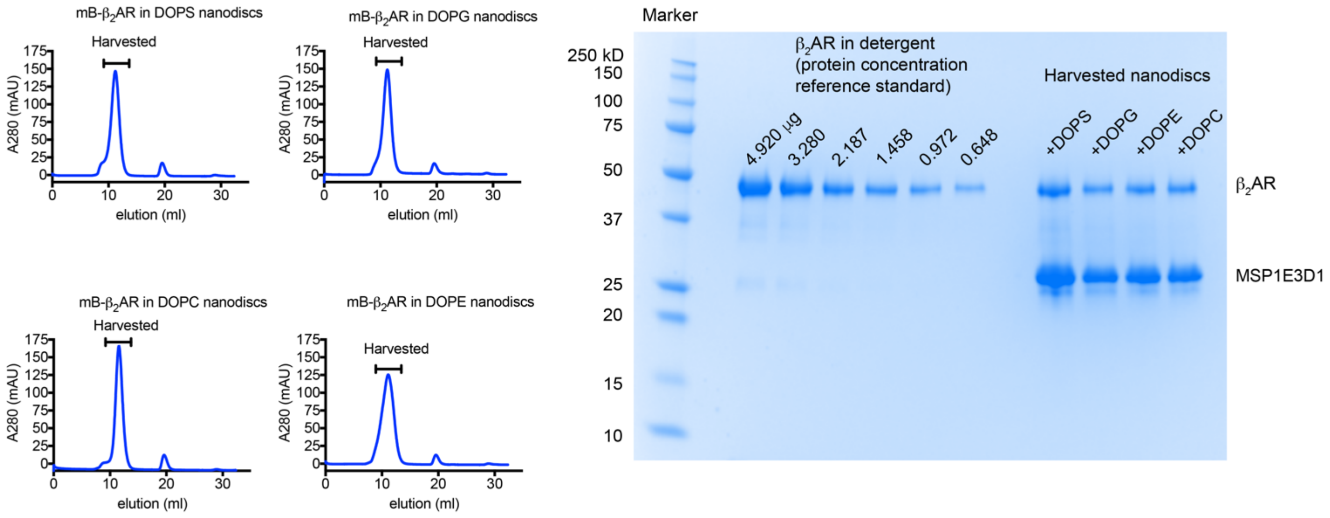

Supplementary figure 5. Left: Size exclusion profiles of nanodisc mB-β_2AR; Right: SDS-PAGE of harvested nanodisc mB-β_2AR after concentration

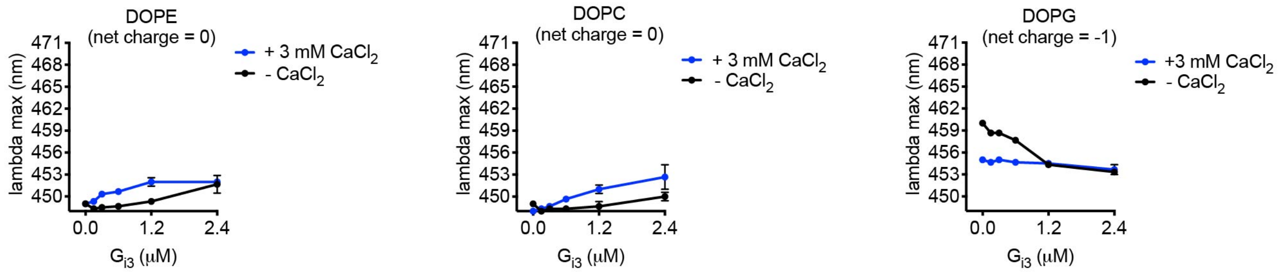

Supplementary figure 6. Effect of Ca^{2+} on basal mB-β_2AR-G_{i3} interaction in bilayers of varying phospholipid composition The effect of G_{i3} concentration on mB-β_2AR fluorescence (+/- 3 mM $CaCl_2$) was examined in DOPE, DOPC, and DOPG nanodisc bilayers in the absence of epinephrine. mB-β_2AR concentration is 100 nM. Data are mean +/- s.e.m of three independent experiments. The net charge of the phospholipid is indicated in parentheses. Source data are provided in the Source Data File.

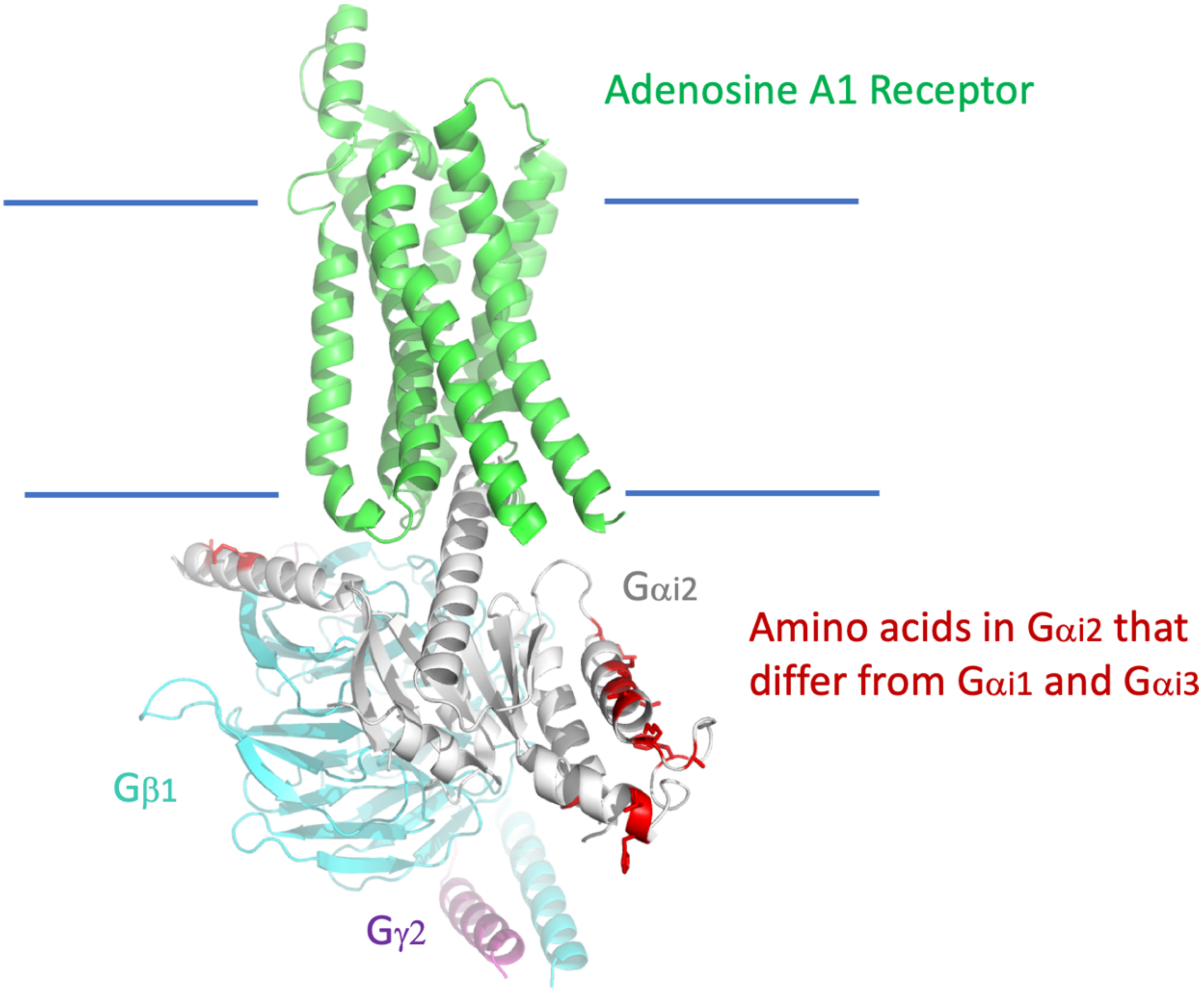

Supplementary figure 7. Cryo-electron microscopy structure of the adenosine A1-G_{i2} complex (PDB: 6D9H). Highlighted in red are amino acids in the Ras domain of $G\alpha_{i2}$ that differ from $G\alpha_{i1}$ and $G\alpha_{i3}$.
