# Supplementary material for: Local membrane charge regulates β2 adrenergic receptor coupling to Gi3
Source: Nat Commun. 2019 May 20;10:2234. doi: 10.1038/s41467-019-10108-0 (PMC6527575; doi:10.1038/s41467-019-10108-0)
Supplement: Supplementary file 2 — Reporting Summary [file 41467_2019_10108_MOESM2_ESM.pdf]

nature research

Corresponding author(s): Brian Kobilka

Reporting Summary

Nature Research wishes to improve the reproducibility of the work that we publish. This form provides structure for consistency and transparency in reporting. For further information on Nature Research policies, see Authors & Referees and the Editorial Policy Checklist.

Statistical parameters

When statistical analyses are reported, confirm that the following items are present in the relevant location (e.g. figure legend, table legend, main text, or Methods section).

n/a	Confirmed	
☐	☒	The exact sample size (n) for each experimental group/condition, given as a discrete number and unit of measurement
☐	☒	An indication of whether measurements were taken from distinct samples or whether the same sample was measured repeatedly
☐	☒	The statistical test(s) used AND whether they are one- or two-sided<br>*Only common tests should be described solely by name; describe more complex techniques in the Methods section.*
☒	☐	A description of all covariates tested
☐	☒	A description of any assumptions or corrections, such as tests of normality and adjustment for multiple comparisons
☐	☒	A full description of the statistics including central tendency (e.g. means) or other basic estimates (e.g. regression coefficient) AND variation (e.g. standard deviation) or associated estimates of uncertainty (e.g. confidence intervals)
☐	☒	For null hypothesis testing, the test statistic (e.g. F, t, r) with confidence intervals, effect sizes, degrees of freedom and P value noted<br>*Give P values as exact values whenever suitable.*
☒	☐	For Bayesian analysis, information on the choice of priors and Markov chain Monte Carlo settings
☒	☐	For hierarchical and complex designs, identification of the appropriate level for tests and full reporting of outcomes
☒	☐	Estimates of effect sizes (e.g. Cohen's d, Pearson's r), indicating how they were calculated
☐	☒	Clearly defined error bars<br>*State explicitly what error bars represent (e.g. SD, SE, CI)*

Our web collection on statistics for biologists may be useful.

Software and code

Policy information about availability of computer code

Data collection	Fluorescence data were collected using FluorEssence v3.8 (Horiba). Luminescence data were collected using SoftMax Pro v6.2.1. (Molecular Devices) and a SpectraMax Paradigm plate reader (Molecular Devices). SDS-PAGE gels stained with Pro-Q Diamond Phosphoprotein Gel Stain (Thermo Fisher Scientific) were scanned with a Typhoon 9410 Imager (GE Healthcare).
Data analysis	Fluorescence and luminesence data were analyzed in Prism 7d (GraphPad). Fluorescence spectra (S1c/R1c) were smoothed using Prism (n=15 neighbors, 2nd order polynomial). To determine the EC50, curves were fit to the “agonist vs. response” model in Prism 7.0 software. Two-way and three-way ANOVA were performed using Graphpad Prism7.0. Structural views and mutant models were generated using PyMOL v 1.8.6.0 (Schrödinger, LLC). Continuum electrostatics models were calculated using the APBS71 plugin (MG Lerner, University of Michigan, Ann Arbor) for PyMOL. Atomic charge and radii were calculated using the online PDB2PQR server (pH 7.4, PARSE force field, hydrogen bond optimization, clash avoidance).

For manuscripts utilizing custom algorithms or software that are central to the research but not yet described in published literature, software must be made available to editors/reviewers upon request. We strongly encourage code deposition in a community repository (e.g. GitHub). See the Nature Research guidelines for submitting code & software for further information.

April 2018

Data

Policy information about availability of data

All manuscripts must include a data availability statement. This statement should provide the following information, where applicable:

- Accession codes, unique identifiers, or web links for publicly available datasets
- A list of figures that have associated raw data
- A description of any restrictions on data availability

The source data underlying Figs. 1a,c-f, 2a-b, 3, 4, 5a-d, and Supplemental Figs. 1a-c, 2a-b, 3a-b, 4, and 6 are provided as a Source Data File. pMSP1E3D1 is available from Addgene (#20066). All PDB files that were analyzed can be obtained from The RCSB Protein Data Bank: PDB 5JQH, 10.2210/pdb5JQH/pdb; PDB 3SN6, 10.2210/pdb3SN6/pdb; PDB 1GP2, 10.2210/pdb1GP2/pdb; PDB 6D9H, 10.2210/pdb6D9H/pdb. All other datasets generated during and analyzed during the study are available from the corresponding author at reasonable request.

Field-specific reporting

Please select the best fit for your research. If you are not sure, read the appropriate sections before making your selection.

☒ Life sciences ☐ Behavioural & social sciences ☐ Ecological, evolutionary & environmental sciences

For a reference copy of the document with all sections, see nature.com/authors/policies/ReportingSummary-flat.pdf

Life sciences study design

All studies must disclose on these points even when the disclosure is negative.

Sample size	No sample size calculations were performed. Experiments were repeated 3+ times to allow calculation of the mean and standard error of the mean.
Data exclusions	No data were excluded from the analyses.
Replication	All attempts at replication were successful.
Randomization	Randomization was not necessary in our study and was not performed.
Blinding	Blinding was not necessary for our study and was not performed.

Reporting for specific materials, systems and methods

Materials & experimental systems

n/a	Involved in the study	
☐	☒	Unique biological materials
☐	☒	Antibodies
☐	☒	Eukaryotic cell lines
☒	☐	Palaeontology
☒	☐	Animals and other organisms
☒	☐	Human research participants

Methods

n/a	Involved in the study	
☒	☐	ChIP-seq
☒	☐	Flow cytometry
☒	☐	MRI-based neuroimaging

Unique biological materials

Policy information about availability of materials

Obtaining unique materials	There are no restrictions on availability.

Antibodies

Antibodies used	anti-FLAG (ATCC #HB-9259, clone 4E11)

Validation	The antibody was used for purification of the beta2 adrenergic receptor. All purifications were successful.

Eukaryotic cell lines

Policy information about cell lines

Cell line source(s)	The Sf9 and Tni cells used were obtained from Expression Systems, Davis, CA. The 4E11 hybridoma cells were obtained from ATCC (#HB-9259).
Authentication	No authentication required.
Mycoplasma contamination	The cell lines were not tested for mycoplasma contamination.
Commonly misidentified lines (See ICLAC register)	Cells are not listed in the database.
